# Metabolic Syndrome as a Risk Factor for Alzheimer’s Disease: A Focus on Insulin Resistance

**DOI:** 10.3390/ijms24054354

**Published:** 2023-02-22

**Authors:** Amaia Ezkurdia, María J. Ramírez, Maite Solas

**Affiliations:** 1 Department of Pharmacology and Toxicology, University of Navarra, 31008 Pamplona, Spain; 2 IdISNA, Navarra Institute for Health Research, 31008 Pamplona, Spain

**Keywords:** hypertension, hyperlipidemia, obesity, diabetes mellitus, insulin resistance, astrocyte

## Abstract

Alzheimer’s disease (AD) is the main type of dementia and is a disease with a profound socioeconomic burden due to the lack of effective treatment. In addition to genetics and environmental factors, AD is highly associated with metabolic syndrome, defined as the combination of hypertension, hyperlipidemia, obesity and type 2 diabetes mellitus (T2DM). Among these risk factors, the connection between AD and T2DM has been deeply studied. It has been suggested that the mechanism linking both conditions is insulin resistance. Insulin is an important hormone that regulates not only peripheral energy homeostasis but also brain functions, such as cognition. Insulin desensitization, therefore, could impact normal brain function increasing the risk of developing neurodegenerative disorders in later life. Paradoxically, it has been demonstrated that decreased neuronal insulin signalling can also have a protective role in aging and protein-aggregation-associated diseases, as is the case in AD. This controversy is fed by studies focused on neuronal insulin signalling. However, the role of insulin action on other brain cell types, such as astrocytes, is still unexplored. Therefore, it is worthwhile exploring the involvement of the astrocytic insulin receptor in cognition, as well as in the onset and/or development of AD.

## 1. Introduction

Alzheimer’s disease (AD) affects 27 million people worldwide and is the most common type of dementia [1]. Indeed, AD makes up 60 to 70% of all dementia cases [2]. The impact on the life of a patient’s family as well as the financial cost to society of the occurrence of the disease is very large, especially due to the fact that, to date, there is no curative treatment for AD [3]. Although AD is a public health issue, to date, only two classes of drugs have been approved for its treatment: cholinesterase enzyme inhibitors (rivastigmine, galantamine and donepezil) and NMDA receptor antagonists (memantine). Even though these two classes of drug show therapeutic effects, they are only effective at treating AD symptoms not preventing the disease [4,5,6]. Regrettably, a low number of clinical trials on AD were launched in the last decade and their outcome was a big failure. A plethora of treatment options are now being intensively studied [6]. However, it is crucial to identify the risk factors involved in the disease as the control of these factors may help to prevent and probably combat the course of the disease. The definition of risk factors can radically change the treatment and diagnosis of AD from conventional approaches towards precision medicine, offering a personalized approach to disease management [7].

The main and earliest manifestation of the disease is the memory impairment that evolves over several years. During the disease progression, intellectual skills deteriorate, behavioural problems and delusion appear, while the patient loses control over essential body functions. Pathologically, the main AD hallmarks are the accumulation of beta-amyloid peptide (Aβ) outside neurons and the hyperphosphorylation and Tau protein aggregation inside neurons [3,6,8,9]. Anatomically, AD patients present a profound brain atrophy, especially at the hippocampal and the neocortical areas [10]. Moreover, in AD brains, reduced levels of acetylcholine, norepinephrine and dopamine have been detected [11,12]. Furthermore, in light of epidemiological and experimental evidence, several pathological events that are not specific to AD have been identified, including brain energy deregulation [13,14,15], synaptic dysfunction [16], oxidative/ER stress [17], mitochondrial alterations [16], autophagy deterioration [18], inflammation [19] and the blood–brain barrier (BBB) [20] and neurovasculature breakdown [21]. In this point, a fundamental question is the following: are these dysfunctions directly connected to the amyloid and Tau pathology? In contrast, are these pathological features occurring parallel to Aβ and Tau? Even more importantly, how are these pathological events induced? Notably, many of those alterations have been directly associated to AD risk factors, pointing towards the importance of the deep study of risk factors that precipitate AD pathology.

This review summarizes our current understanding of the impact of metabolic syndrome in the development of dementia, especially AD. We mainly focus on mechanisms and clinical outcomes of type 2 diabetes mellitus (T2DM), address the apparent paradox of how impaired neuronal insulin can protect from the development of neurodegenerative disorders and propose astrocytic insulin signalling as a new target to explore.

## 2. Association between Metabolic Syndrome and Alzheimer’s Disease

Genetic variants as well as environmental or non-genetic factors have been linked to the onset and/or the progression of AD [22,23,24,25,26,27,28]. Some of the most studied environmental risk factors include aging, cardiovascular disease [29,30,31], T2DM [26,32], obesity [33], depression, dyslipidaemia [23], substance abuse or smoking. These risk factors may lead to the above-mentioned pathogenic features, i.e., reduced glucose utilization, oxidative stress, chronic inflammation, mitochondrial dysfunction or brain energy metabolism breakdown among others, that could cause or aggravate AD development [33,34,35,36].

**Figure 1:**
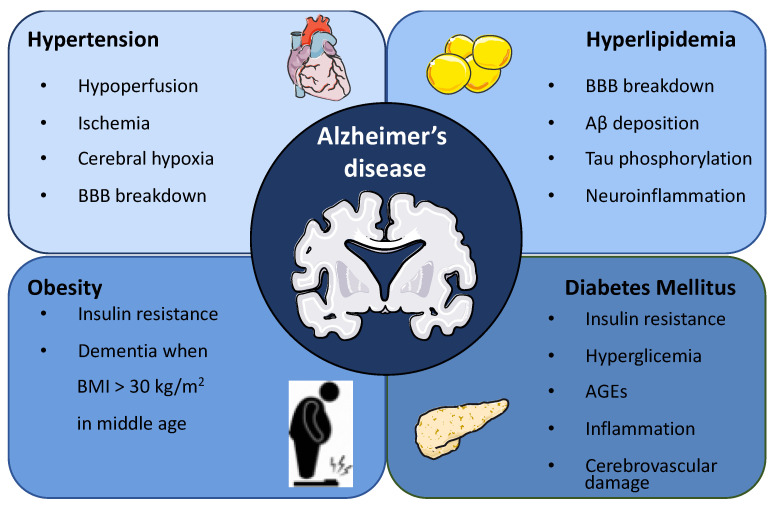
Metabolic syndrome as a risk factor for Alzheimer’s disease. Metabolic syndrome is defined as the coexistence of obesity, hypertension, hyperlipidemia and diabetes. Specifically, hypertension alters the vascular walls leading to hypoperfusion, ischemia and cerebral hypoxia, triggering AD development. Hyperlipidemia compromises BBB integrity, increases Aβ peptide deposition, promotes Tau hyperphosphorylation and induces neuroinflammation compatible with AD. The involvement of obesity as a risk factor for AD development is still under debate, although some studies have demonstrated that obesity in middle age is a risk factor for dementia. The association between diabetes mellitus (DM) and AD has been extensively studied. The suggested mechanisms underlying this association include, among others, insulin resistance, insulin receptor impairment, hyperglycaemia-induced toxicity, advanced glycation end products (AGEs)-induced adverse effects, inflammation and cerebrovascular damage.

Environmental AD risk factors rarely exist alone. Obesity, hypertension, dyslipidemia and diabetes often coexist, and their effects are hard to tease apart. Usually, most of the studies focus on one factor, neglecting the possibility that different risk factors could increase the risk of AD in an additive or synergistic manner. Therefore, a more accurate approach is the study of a cluster of risk factors such as the metabolic syndrome, defined as the coexistence of obesity, hypertension, hyperlipidemia and diabetes (Figure 1) [33]. The association of these risk factors to AD development is described in the following subsections.

### 2.1. Hypertension

It has been suggested that the association between hypertension and AD occurs through cerebrovascular disease. Specifically, hypertension alters the vascular walls leading to hypoperfusion, ischemia and cerebral hypoxia, triggering AD development. According to the literature, cerebral ischemia precipitates the accumulation of Aβ and induces the expression of presenilin that is involved in Aβ synthesis. Moreover, hypertension promotes BBB breakdown, a feature intimately linked to AD pathology [37].

According to epidemiological studies, an association between hypertension and dementia has been described [38]. Notably, the most compelling evidence is obtained when hypertension is present in middle age and precipitates AD and vascular dementia 15 to 20 years later [39].

### 2.2. Hyperlipidemia

Previous studies have already demonstrated that patients with AD show 10% higher cholesterol levels compared to healthy individuals [40], proposing cholesterol as a risk factor for the development of AD.

Elevated levels of cholesterol compromises BBB integrity [41], consequently increasing AD risk. Moreover, investigations in experimental animal models have shown that hypercholesterolemia increases Aβ peptide deposition, Tau hyperphosphorylation, neuroinflammation, cognitive deficiency, cholinergic neuron dysfunction and cerebral microhaemorrhages and is compatible with AD [42,43].

Observational studies have pointed out that statin users show a reduced incidence of AD or even an improvement in the disease progression [44,45,46]. However, most clinical studies have not demonstrated the efficacy of statins against AD onset and/or progression at various stages of the disease [47,48,49,50,51], in contrast to the study conducted by Song et al. [52] that observed a lower risk of AD in statin users.

Apart from cholesterol, chronic high free fatty acid levels have been shown to induce pernicious outcomes, including low-grade inflammation that could lead to insulin resistance. Although the capacity of fatty acids to pass through the BBB is limited [53], PET studies have demonstrated fatty acids uptake by the brain [54]. Notably, metabolic syndrome induces the brain’s uptake and accumulation of fatty acids that can be reversed by weight reduction [54]. Interestingly, it has been extensively reported that the exposure to a high fat diet promotes AD pathogenesis, and diets enriched in polyunsaturated fatty acids such as docosahexaenoic acid (DHA) show a protective effect against AD [55]. Saturated fatty acids could promote the brain inflammatory response through TLR4 activation [56]. Indeed, the loss of function of TLR4 protects against high-fat-diet-induced deleterious effects [56]. Moreover, the molecular link between high levels of fatty acids and AD could be beta-amyloid and Tau, as free fatty acids have been shown to stimulate the assembly of both amyloid and tau filaments in vitro [57] leading to cognitive dysfunction.

### 2.3. Obesity

The prevalence of obesity and overweight is exponentially increasing, with an estimation of the existence of 1.35 billion overweight and 573 million obese people around the world by 2030 [58]. The risk of suffering dementia is significantly increased upon obese conditions. Indeed, in a longitudinal study where participant’s sagittal abdominal diameter was measured, a larger diameter was directly correlated with nearly a three-fold risk of developing dementia [59]. In the same line, another study demonstrated that a lower hippocampal volume is observed in subjects with higher waist–hip ratio [60]. The connection between midlife obesity and the risk of suffering dementia in the elderly has been investigated by several authors [59,61,62,63,64]. In the study performed by Xu et al., midlife overweight (BMI > 25–30) and obese (BMI > 30) individuals showed a higher dementia probability [65]. Another cohort study demonstrated that while obesity in midlife significantly increases the risk of suffering dementia later in life, this risk is decreased when obesity occurs in old age [66]. Remarkably, a cohort projection model based on an Australian population demonstrated that if midlife obesity is decreased by 20%, dementia in aging could be lowered by 10% [67]. In contrast with all the above-mentioned studies that suggests a direct link between midlife obesity and late life dementia, a cohort study showed that being underweight in middle age carries an increased risk of dementia in later life [68]. Based on these conflicting results, the possible connection between obesity and dementia needs a deeper investigation. It is worth mentioning that it has been proposed that the linking mechanism between obesity and AD could be obesity-induced insulin resistance.

### 2.4. Diabetes Mellitus

The association between diabetes mellitus (DM) and AD has been extensively studied, with most research showing a clear link between T2DM and a higher risk of developing AD. The suggested mechanisms underlying this association include, among others, insulin deficiency, insulin resistance, insulin receptor impairment, hyperglycaemia-induced toxicity, advanced glycation end products (AGEs)-induced adverse effects, inflammation and cerebrovascular damage [69].

The connection between T2DM and AD has been studied using different approaches, extensively described in Section 4 of the present review. For instance, post-mortem brains of AD patients present a deregulation of the insulin receptor (IR) intracellular signalling [70,71,72]. The exposition of AD transgenic mouse models to a high fat and/or high sugar diet exacerbates the AD pathology [73,74,75,76,77]. Upon antidiabetic treatment, a slight cognitive improvement, as well as the alleviation of inflammation, apoptosis and synaptic failure, has been described in human and AD mouse models [78,79]. Finally, DM murine models recapitulate the alteration in glucose metabolism, IR signalling, neuroinflammation, Tau hyperphosphorylation and Aβ aberrant processing characteristic of AD pathology [80,81]. However, the extensive literature, employing very different approaches, has produced conflicting results based on variables such as the model used, the type of diet, the duration of the study, etc. Thus, it is still difficult to have a definite idea of how T2DM is linked to AD. This conundrum will be discussed below.

## 3. Insulin Action in the Brain

Traditionally, it has been thought that the brain is an insulin-insensitive organ. However, during the last few years, new evidence showing a high concentration of insulin in brain extracts, and expression of IR throughout the brain, supports that it is actually an organ that responds to this hormone [82]. Insulin is an important regulator of glucose homeostasis and metabolism. Nevertheless, its role in the central nervous system is a field that is not yet fully known. It has been suggested that it can modulate central functions such as feeding, depression and cognitive behaviour [83].

### 3.1. Insulin Transport and Regional Distribution in the Brain

It has been proposed that insulin reaches the brain in two ways: across the BBB or synthesizing de novo in the brain. However, whether insulin can be locally synthesized in the brain remains under debate since the synthesis of insulin in the central nervous system (CNS) seems to depend on the animal species under study. In vitro experiments have shown that insulin can be synthesized and secreted by rabbit CNS neuronal cells in culture but not in glia [84]. Moreover, the expression of insulin mRNA has been detected in rat and mouse brains [85,86,87]. In humans, peptide C (by-product of insulin synthesis) has also been observed in several brain regions [88,89]. However, these studies have been questioned and others have not been able to demonstrate the presence of insulin mRNA in appreciable amounts in the brain. In general, there is a lack of data suggesting that the human brain produces and secretes significant amounts of insulin locally, and it is widely believed that the insulin found in the brain parenchyma comes primarily from the pancreas [90].

Insulin enters the brain across the capillary endothelial cells of the BBB via selective, saturable transport. Insulin levels in cerebrospinal fluid (CSF) are much lower than in plasma. Even so, there is a connection between both since the increase in peripheral insulin concentration acutely increases concentration in the brain and CSF, indicating that most of the insulin that reaches the brain derives from circulating pancreatic insulin [91,92]. This transport can be modulated by a vast array of factors such as DM, inflammation, obesity and circulating triglyceride levels.

IRs are widely distributed throughout the CNS, following a selective regional pattern. In rodents, the highest IR expression is found in the olfactory bulb, hypothalamus, cerebral cortex, hippocampus and cerebellum [93,94]. Thus, IRs are present in brain areas related to glucose and energy homeostasis as well as cognitive processes (i.e., the hypothalamus and the cortex/hippocampus, respectively) [95].

### 3.2. Brain Insulin Actions in Peripheral Energy Homeostasis

Studies that have been carried out on insulin and IRs in the brain demonstrate a key role of central insulin signalling in the regulation of peripheral functions [96]. Insulin acts within the hypothalamus to regulate various processes of metabolism such as energy homeostasis (body weight and food intake), glucose metabolism and the regulation of reproduction.

#### 3.2.1. Energy Homeostasis

Insulin acts in two neuronal populations in the arcuate nucleus of the hypothalamus (ARC): the orexigenic neurons (coexpressing neuropeptide Y, NPY, and agouti-related peptide, AgRP) and the anorexigenic neurons (which produce proopiomelanocortin, POMC, and cocaine- and amphetamine-related transcript, CART). In the ARC, the role of insulin signalling on neuronal activity seems to be complex, as it acts by hyperpolarizing both anorexigenic POMC neurons and orexigenic AgRP/NPY neurons [97].

Intracerebrovascular administration of insulin has an anorexigenic effect; it inhibits food intake, resulting in a reduction in body weight. By contrast, inhibition of insulin signalling in the brain has an orexigenic effect, it increases food intake, and as a result increases the body weight [98].

At a cellular level, insulin regulates transcriptional events, by inducing *Pomc* transcription, and decreasing *Agrp* expression, resulting in an increase in the anorexigenic tone upon feeding [99]. Furthermore, in the postprandial state, the precursor protein POMC is converted into α-melanocyte-stimulating hormone (α-MSH) and released from POMC neuron synaptic endings located in ARC to paraventricular hypothalamic nucleus (PNV) where it activates melanocortin 3 and 4 receptors (MC3/4-R). Melanocortin receptor (MC-R) activation decreases food intake, increases energy expenditure and regulates glucose metabolism [100].

#### 3.2.2. Glucose Metabolism

In addition to its anorexigenic effect, insulin plays an important role in the CNS-dependent regulation of peripheral glucose metabolism. Central insulin infusion results in a decrease in hepatic glucose production (HGP) [101,102]. This effect is not observed in neuronal IR knockout mice (NIRKO mice), nor in AgRP-restricted IR knockout mice, suggesting that insulin action in AgRP-expressing neurons is required for the suppression of HGP [101].

The anorexigenic effect and ability to suppress HGP of insulin are mediated by the phosphatidylinositol 3-kinase (PI3K)/protein kinase B (AKT) pathway, mainly through forkhead box protein O1 (FOXO1) and the activation of the adenosine triphosphate (ATP)-dependent potassium channel [95,97,98]. Nuclear exclusion of FOXO1 increases the transcription of POMC and decreases AgRP and G-protein-coupled receptor 17 (GPCR17). GPCR17 might control food intake and HGP by the modulation of ion channel activity and the resultant increase in orexigenic neuropeptide release [103]. Insulin also acts via activation of the ATP-dependent potassium channel, leading to neuronal hyperpolarization and inhibiting the secretion of neuropeptides [95,97,98].

### 3.3. Brain Insulin Actions in CNS Functions

It has been shown that insulin is involved in the CNS and brain function. However, the role it can play is not yet fully understood. Some studies pointed out the effects of insulin on learning and memory due to insulin and IRs presence in the hippocampus and cerebral cortex [104]. Indeed, it has been proposed that insulin regulates cognition modulating synaptic plasticity, synapse density and neurotransmission, and even by regulating adult neurogenesis [105].

#### 3.3.1. Neuronal Plasticity

Neuronal plasticity encompasses all the processes through which neurons adapt to changes in functional demands, and insulin-like peptides (ILPs) act by modulating these processes [105]. ILPs affect synaptic efficacy by modulating various components of the synapse and their expression. In the past decades electrophysiology studies have revealed the importance of synaptic signalling in long-term potentiation (LTP) and long-term depression (LTD) [106]. In vitro experiments using cultured neurons showed that ILPs could modulate LTP and LTD, regulating the synthesis of glutamate and GABA receptor subunits and changing the phosphorylation of glutamate receptor subunits [107,108].

Insulin can regulate the AMPA receptor that underlies synaptic transmission and synaptic plasticity [109]. It is seen that insulin induces glutamatergic AMPA receptor internalization leading to LTD [110]. Moreover, LTD induced by AMPA receptor internalization has been demonstrated to regulate the extinction of the fear-conditioned memory process [111], thereby linking the insulin-involved synaptic activities to cognition.

Conversely, the downregulation of hippocampal IR function impairs LTP and spatial memory [112]. Insulin also potentiates NMDA receptor activity by expressing NMDA receptors to the cell surface [113] and through NMDA receptor phosphorylation [114], processes that may induce long-lasting changes.

In addition to AMPA and NMDA receptors, insulin has been shown to be a regulator of GABA(A) receptors. Specifically, it increases inhibitory synaptic transmission by recruiting β2 subunits of GABA(A) receptors at the postsynaptic level [108]. Moreover, ILPs contribute to modulating synaptic strength by regulating the synthesis and release of neurotransmitters such as acetylcholine in the hippocampus [105].

#### 3.3.2. Dendritic Arbor Development

PI3K/AKT and mitogen-activated protein kinase (MAPK) pathways include components that have been implicated in dendritic arbor formation dynamics. IRSp53, an IR substrate localized in the postsynaptic density, has been hypothesized to interact and stabilize the proteins of the cytoskeleton in the postsynaptic density region [115]. Moreover, in cell cultures, it has been demonstrated that increased expression of IRSp53 promotes dendritic arbor development [116], while RNA-interference-treated cells against IRSp53 showed the opposite effect [117].

#### 3.3.3. Neuronal Survival

It is seen that IR signalling promotes cell survival under oxygen and glucose deprivation in rat hippocampal cell culture studies. Although the exact mechanism remains unclear, the authors proposed increased GABA signalling as a potential mechanism of insulin action to protect neurons from oxygen–glucose-deprivation-induced cell death [118].

#### 3.3.4. Learning and Memory

Intranasal administration of insulin has shown to improve memory and learning in rats. Other studies in healthy humans demonstrated that CNS insulin administration via the intranasal route have beneficial cognitive effects [119,120], indeed this improvement could even be intensified by the administration of rapid-acting insulin analogue, named insulin aspart [121]. Furthermore, systemic infusion of insulin in healthy humans improved verbal memory and selective attention [122]. Gene expression of IR has been increased in the hippocampal dentate gyrus and CA1 region of the rat after spatial memory training [104].

Although the mechanisms involved in these processes are not fully known, it is seen that the PI3K pathway is involved since the central administration of insulin increases memory in a PI3K-dependent manner [123]. Nevertheless, NIRKO mice showed unaltered spatial learning during a Morris Water Maze task, indicating that the insulin pathway does not play a primary role in memory formation [124].

## 4. Controversial Role of Insulin Alterations in Alzheimer’s Disease

Based on the above-mentioned important role of insulin in cognitive functions, the interaction between DM and AD has gained great attention in the past two decades [36]. Indeed, not only insulin deficiency but also insulin resistance has pernicious consequences on the brain [125,126].

There are multiple plausible mechanisms that could explain the connection between insulin resistance and AD. Insulin resistance is defined by the diminished response to insulin that occurs as a response to certain genetic and/or environmental factors [127]. Upon an insulin resistance state, insulin secretion from the pancreas is increased in an attempt to achieve normoglycemia. This hyperinsulinemia leads to a vicious cycle that worsens the insulin resistance [128,129] and could be implicated in AD pathogenesis [34,130,131]. Peripheral hyperinsulinemia can also affect its own transport across the BBB [125,132]. Once in the brain, insulin is degraded by the insulin-degrading enzyme (IDE), an enzyme able to degrade the Aβ protein as well [125,133]. Notably, insulin and Aβ compete for the binding to the IDE, the insulin affinity being higher compared to Aβ [133]. Therefore, in a state of hyperinsulinemia, insulin binds the IDE leading to Aβ protein accumulation [125,133]. Moreover, Aβ protein levels in the extracellular space are increased upon high insulin levels [133]. Interestingly, Aβ binds and avoids the insulin binding to IR, exacerbating insulin resistance [34,36,133,134]. In this line, considering the close link between Aβ and impaired insulin signalling, it is not surprising that impaired cerebral glucose metabolism usually precedes AD signs and symptoms by several years [35,135,136]. Notably, hyperglycaemia as well as insulin resistance induces cognitive deficiencies, one of the core symptoms of AD [137].

Insulin resistance has also been associated to Tau hyperphosphorylation [138]. Glycogen synthase kinase 3 (GSK3β) is a metalloprotease that acts by phosphorylating the Tau protein, which, when hyperphosphorylated, precipitates and accumulates in the form of neurofibrillary tangles (NFTs). Insulin is an inhibitor of GSK3β, thus preventing Tau from being phosphorylated. Therefore, it is suggested that in situations of insulin resistance, the activity of GSK3β is increased and leads to the phosphorylation of Tau and formation of NFTs [139,140].

Furthermore, insulin resistance reduces acetylcholine levels in the brain leading to cholinergic perturbations, which are largely involved in AD pathology [11,141,142].

The chronic state of hyperinsulinemia interferes with the saturable transport of insulin through the BBB. Thus, at some point, brain insulin resistance could be accompanied by reduced insulin levels at the CNS [36,125,132] and this brain insulin deficiency has also been associated with AD onset and progression [125,143]. In summary, AD can be considered a situation in which insulin deficiency as well as insulin resistance are profoundly implicated [144,145].

Other possible mechanisms linking DM and AD have also been described, such as chronic inflammation and oxidative stress [25,34,146]. High levels of inflammatory cytokines, such as interleukin-1 β, interleukin-6 and interferon gamma are located close to Aβ plaques and macrophage cells suggesting the important role of neuroinflammation in the pathology of AD. Peripheral hyperinsulinemia leads to the maintenance of inflammation through its inhibition of AMP-activated protein kinase [147]. These inflammatory processes could mediate the relationship between T2DM and AD.

Another proposed pathogenic pathway is the alteration of gut microbiota and the subsequent disruption of the gut–brain axis that occurs upon insulin resistance [148,149,150,151,152,153]. Gut microbiota disturbance has been associated with AD [154,155].

Finally, another plausible mechanism linking DM and AD could be cerebrovascular diseases (CVD), as insulin resistance and the subsequent hyperinsulinemia are involved in CVD [156,157,158,159] that could eventually lead to AD pathology.

In the light of all those shared mechanisms between DM and dementia, AD has been described as type 3 diabetes [160]. However, this concept is still under intense debate, as many studies in the literature do not support this idea. For instance, Hardy et al. tested for genetic correlation between T2DM and AD using a genome-wide association study (GWAS), without finding convincing evidence for a genetic overlap between both disorders. Specifically, none of the single nucleotide polymorphisms (SNP) found in one disease were relevant in the other disease genome [161].

**Figure 2:**
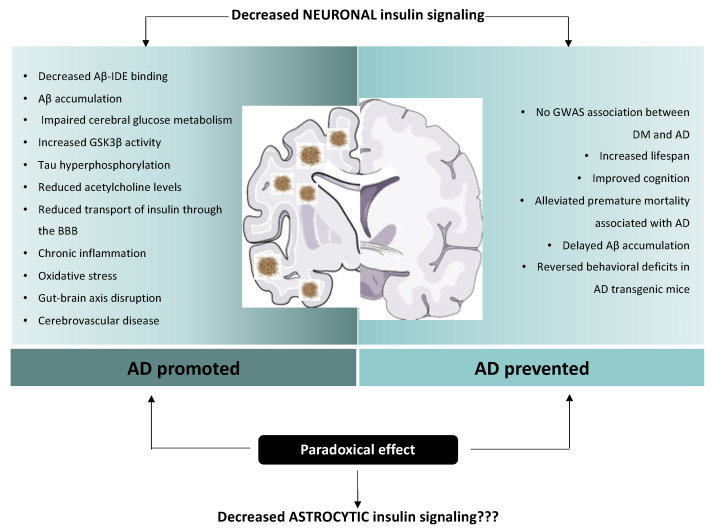
Paradoxical effect of insulin on Alzheimer’s disease etiopathology. Multiple plausible mechanisms have been proposed to explain the connection between insulin resistance and AD. Insulin and Aβ compete for the binding to IDE, leading to Aβ protein accumulation. Moreover, cerebral glucose metabolism usually precedes AD signs. In situations of insulin resistance, the activity of GSK3β is increased and leads to the hyperphosphorylation of Tau. Furthermore, insulin resistance reduces acetylcholine levels in the brain. Chronic state of hyperinsulinemia interferes with the saturable transport of insulin through the BBB. Other possible mechanisms linking DM and AD have also been described, such as chronic inflammation, oxidative stress, disruption of the gut–brain axis and cerebrovascular diseases. Strikingly, a plausible protective role of diminished neuronal insulin signalling has also been proposed. It has been seen that decreased neuronal insulin signalling has a beneficial effect on lifespan regulation, and can even delay age-related processes. Decreased insulin signalling has also been associated with reduced premature mortality associated with AD and delayed Aβ accumulation. Due to the paradoxical effects of insulin signalling, the current state of our understanding is insufficient to differentiate clearly between the beneficial vs. negative impact on aging and neurodegenerative diseases of reduced insulin signalling. Notably, this debate is fed by results obtained from studies focused exclusively on neuronal insulin signalling; however, the role of insulin action on other brain cell types, such as astrocytes, is still unexplored.

Moreover, there are conflicting findings on the neuroprotective effects of the insulin signalling pathway (Figure 2). Indeed, it has been seen that decreased neuronal insulin signalling has a beneficial effect on lifespan regulation, and even delay age-related processes.

In *Caenorhabditis elegans*, the human insulin and insulin-like growth factor 1 (IGF-1) receptors are codified by a single ortholog, DAF-2 [162]. Dillin et al. showed that adult-specific RNAi knockdown of DAF-2 increases lifespan [163]. In a similar manner, the group led by Ewald CY found that using an auxin-inducible degradation system, the depletion of DAF-2 at post-reproduction stages resulted in a 70–135% lifespan extension without inducing adverse phenotypes such as growth retardation, germline shrinkage, egg retention or reduced brood size. Impressively, at geriatric ages, auxin-mediated depletion of DAF-2 protein resulted in a doubling of lifespan [164].

In mammals, it is seen that midlife administration of IGF-1 receptor monoclonal antibodies is sufficient to improve health and increase lifespan, preferentially in females [165]. Furthermore, loss-of-function mutations in genes encoding for insulin receptor substrate 1 and 2 (IRS1/2) [166,167] and IGF-1 receptor [167,168] increased longevity in rodents. Decreased insulin signalling has also been associated with an improvement in age-related intracellular and behavioural events that are related to neurodegenerative disorders, and thus cognition [95]. In particular, IGF-1 deficiency in a genetic model of AD reversed premature mortality associated with AD and delayed Aβ accumulation [167]. Additionally, deletion of IRS-2 reduces Aβ deposition and rescues behavioural deficits in APP transgenic mice [169].

Due to the paradoxical effects of insulin signalling, the current state of our understanding is insufficient to differentiate clearly between the beneficial vs. negative impact on aging and neurodegenerative diseases of reduced insulin signalling (Figure 2). Notably, this debate is fed by results obtained from studies focused exclusively on neuronal insulin signalling; however, the role of insulin action on other brain cell types, such as astrocytes, is still unexplored. Indeed, the possibility that insulin signalling in astrocytes plays a functional role in cognitive performance and AD has never been studied. Therefore, it is worthwhile exploring the involvement of astrocytic IR in cognition, as well as in the onset and/or development of neuropsychopathologies.

## 5. Role of Astrocytic Insulin Receptor

There is increasing evidence that non-neuronal cells contribute to the onset, progression and pathology of diverse neurodegenerative diseases. Interestingly, astrocytes are the most abundant cells in the brain and they are closely associated to neurons [170]. They provide an adequate microenvironment to guarantee the correct neuronal function, including the control and formation of synaptic plasticity. They participate in neurogenesis, regulation of the vascular tone of the brain, maintenance of the BBB and regulation of energy homeostasis [171]. Moreover, they can sense neurotransmitters and respond with increases in intracellular calcium level, and they can also release gliotransmitters to neurons [172].

Astrocytes are altered in neurodegenerative disorders [173,174], where dysregulation of astrocyte calcium activity, glutamate uptake and mitochondrial respiration could be seen, leading to damage to neural health [175,176].

### 5.1. Astrocytic Function in AD

For years, Aβ plaques have been considered the main pathological hallmark of AD and responsible for triggering astrocytic reactivity, as some studies showed a higher density of reactive astrocytes around plaques [177].

The glymphatic system participates in a correct fluid clearance where interstitial fluid is cleared along paravenous routes [178], and astrocytes participate actively in this process. They contain high levels of aquaporin 4 (AQ4), which facilitates astrocyte clearance of factors towards the systemic circulation. In AD, there is a downregulation of AQ4 levels, which can result in decreased clearance of Aβ and the subsequent possible protein accumulation [179]. Indeed, AQ4 knockout in an AD mouse model increased Aβ accumulation in the brain, and resulted in cognitive impairment [180].

Neurons are isolated from systemic circulation by the BBB and rely on astrocytes to obtain metabolic substrates [181]. A well described mechanism is the astrocyte–neuron lactate shuttle (ANLS) [182]. As known, glucose is converted to lactate in astrocytes and then exported to neurons for energy use or memory function among other things [183]. Based on the evidence that the energy homeostasis required for synaptic activity is altered in neurodegeneration, a central role of astrocytes in AD is suggested.

In physiological conditions, calcium signalling in astrocytes is associated with multiple processes, such as neuronal activity or the response to synaptic stimulation [184]. Ca^2+^ dynamics in astrocytes were studied in a three-dimensional two-photon imaging approach. It was found that Ca^2+^ activity in astrocyte processes and endfeet displayed frequent fast activity, whereas the soma was infrequently active. Astrocytes responded locally to minimal axonal firing with time-correlated Ca^2+^ spots [185]. Dysregulation of astrocyte calcium signalling has been observed in mouse models of AD before the appearance of astrogliosis, suggesting that this alteration occurs early in the pathology. A study performed in the APP-PS1 mouse model of AD showed that chronic blockade of the calcium channel TRPA1 was sufficient to normalize astrocytic activity, avoid perisynaptic astrocytic process withdrawal, prevent neuronal dysfunction and preserve structural synaptic integrity, thus preserving spatial working memory in that model. In that study, they also showed that brief exposure of hippocampal slices to Aβ oligomers failed to induce synaptic dysfunction when calcium level elevation was blocked in astrocytes, suggesting that astrocyte calcium signalling is associated with synapses [186].

Astrocytes participate in neurotransmitter uptake and recycling. The main excitatory neurotransmitter in the brain is glutamate, which is taken up by transporters localized in astrocytes [187]. Neuronal death caused by excessive postsynaptic receptor activity at glutamatergic synapses has been proposed to explain the pathogenesis of AD [188]. Neuronal death leads to alteration at synapses, where astrocytes could contribute. The overactivation of extra synaptic glutamatergic NMDA receptors, which is a consequence of glutamate release from astrocytes after exposure to Aβ, connects glutamate dyshomeostasis and the loss of synapses observed in AD [189,190]. Not only the release of glutamate from astrocytes, but also the release of ATP [191], D-serine [186] and GABA [192] are increased in AD, suggesting that astrocytes contribute to synaptic alteration and cognitive performance.

### 5.2. Key Role of Astrocytic Insulin Signalling in AD

The role of insulin in neurons has been widely studied. Nevertheless, the role of insulin action in astrocytes and neurobehaviours remains less well studied.

In 2016, García-Cáceres et al. reported that insulin signalling in astrocytes co-regulates behavioural responses and metabolic processes via the regulation of glucose uptake across the BBB. On the one hand, astrocytic IR ablation affects hypothalamic astrocyte morphology, mitochondrial function and circuit connectivity. On the other hand, it reduces glucose-induced activation of hypothalamic POMC neurons and impairs physiological responses to changes in glucose availability [171].

In 2018, Cai et al. demonstrated for the first time that a mice model with IR knockout in astrocytes showed anxiety- and depressive-like behaviours. Mechanistically, the loss of insulin signalling in astrocytes impairs the tyrosine phosphorylation of Munc18c and appears to decrease the exocytosis of ATP from astrocytes, leading to a reduction in dopamine release from the nucleus accumbens among others, affecting neuronal circuits involved in cognition and mood [193].

It is also suggested that insulin action in astrocytes plays an important role in the regulation of cognition and mood [194,195]. APP/PS1 mice showed elevated basal and stimulus-evoked hippocampal glutamate release, astrogliosis and impaired insulin sensitivity that was exacerbated in a high fat diet (HFD). The elevated astrogliotic response surrounding the plaques in APP/PS1 HFD mice could be a compensatory mechanism to control Aβ accumulation [196]. Likewise, streptozotocin treatment in a rat astrocytoma cell line, resulted in an IR mRNA decrease and protein expression, and induced neuroinflammation and amyloidogenesis [197]. Additionally, other studies support that astrocyte-released insulin and IGF-1 protect neurons from synaptotoxic Aβ peptide oligomers [198].

Although astrocytes actively coordinate brain energy homeostasis, and there is increasing evidence of the connection between astrocytic insulin signalling and pathophysiological mechanisms related with AD, many questions still have to be answered [199].

## 6. Concluding Remarks

AD is a dramatic disease without any effective treatment. In this scenario, it is essential to tackle the modifiable risk factors such as obesity, diabetes, hypertension and dyslipidemia (i.e., metabolic syndrome) in an attempt to prevent the onset and progression of AD and improve the quality of life of patients. In this context, DM has gained central attention in the last decades. However, acting on neuronal insulin signalling has controversial effects on the pathogenesis of AD. Therefore, since no current drug intervention can modify the pathophysiological mechanisms related to the development of this devastating disease, in order to find an effective treatment, the focus should move towards other brain cells such as astrocytes, as they could be the main actors in the pathology of AD.

In this regard, targeting astrocytic insulin signalling may be more relevant as a way to treat AD because this may not only halt disease progression but also bypass the paradoxical actions of neuronal insulin. In this sense, it is crucial to clarify these discrepancies regarding the role of neuronal vs. astrocytic insulin signalling in brain function and to delineate the exact mechanisms that lead independently and synergistically to the onset of T2DM and AD. This will ultimately open new avenues in the development of more efficient preventive and therapeutic strategies.

## Data Availability

Not applicable.
